# Natural Variation in the Yeast Glucose-Signaling Network Reveals a New Role for the Mig3p Transcription Factor

**DOI:** 10.1534/g3.112.004127

**Published:** 2012-12-01

**Authors:** Jeffrey A. Lewis, Audrey P. Gasch

**Affiliations:** * Laboratory of Genetics, University of Wisconsin-Madison, Wisconsin 53706; † Great Lakes Bioenergy Research Center, University of Wisconsin-Madison, Wisconsin 53706; ‡ Genome Center of Wisconsin, University of Wisconsin-Madison, Wisconsin 53706

**Keywords:** *Saccharomyces cerevisiae*, glucose signaling, gene expression, natural variation, environmental stress

## Abstract

The Crabtree effect, in which fermentative metabolism is preferred at the expense of respiration, is a hallmark of budding yeast’s glucose response and a model for the Warburg effect in human tumors. While the glucose-responsive transcriptional repressors Mig1p and Mig2p play well-characterized roles in the Crabtree effect, little function for the related Mig3p transcription factor has been uncovered, despite numerous investigations of laboratory yeast strains. Here we studied a wild isolate of *Saccharomyces cerevisiae* to uncover a critical role for Mig3p that has been lost in S288c-derived laboratory strains. We found that Mig3p affects the expression of hundreds of glucose-responsive genes in the oak strain YPS163, both during growth under standard conditions and upon ethanol treatment. Our results suggest that Mig3p may act as a multifunctional activator/repressor that plays separate roles under standard *vs.* stress conditions and that this function has been largely lost in the lab strains. Population analysis suggests that the lab strain and several wild strains harbor mutations that diminish Mig3p function. Thus, by expanding our attention to multiple genetic backgrounds, we have uncovered an important missing link in a key metabolic response.

One of the great challenges in the post-genomic age is to identify functions for uncharacterized genes to generate an integrated view of cellular physiology. Even in the well-studied model organism *Saccharomyces cerevisiae*, more than 1100 genes (∼18%) have unknown functions, with many more genes remaining poorly characterized (Pena-Castillo and Hughes 2007). One explanation for these missing links is that laboratory conditions do not mimic the complex environments found in nature, under which specialized gene functions might be revealed. Another possibility is that gene functions vary significantly across different genetic backgrounds. For example, Dowell *et al.* showed that several yeast genes are essential in one strain background but dispensable for viability in another strain (Dowell *et al.* 2010). The majority of yeast studies focus on a small number of related laboratory strains, which have been inadvertently selected for robust laboratory growth. The focus on a handful of genetic backgrounds of this important model organism may therefore provide an incomplete view of eukaryotic physiology.

We previously showed that the S288c lab strain has an aberrant response to ethanol, which we leveraged to identify new genes and processes involved in ethanol tolerance (Lewis *et al.* 2010). *MIG3* was uncovered in that study as a gene that was differentially expressed across strains responding to ethanol (with strong ethanol-dependent repression in the lab strain but not in wild strains). Mig3p is a Cys_2_His_2_ zinc finger protein that shares sequence similarity with two other transcription factors, Mig1p and Mig2p. Although Mig2p and Mig3p are more similar to each other, all three transcription factors share extensive identity (>70%) in their DNA binding domains, and they bind to nearly identical sequences *in vitro* (Badis *et al.* 2008; Hazbun and Fields 2002; Lutfiyya *et al.* 1998). Mig1p and Mig2p play well-defined roles in glucose-responsive repression of genes involved in gluconeogenesis, aerobic respiration, and alternative carbon-source utilization (Lutfiyya *et al.* 1998; Westholm *et al.* 2008). However, little related function has been uncovered for Mig3p. Several microarray experiments concluded that Mig3p plays no significant role in glucose repression (Lutfiyya *et al.* 1998; Westholm *et al.* 2008). A separate study implicated Mig3p in the transcriptional response to DNA damage (Dubacq *et al.* 2004), while another study found that Mig3p overexpression could confer arsenic resistance (Takahashi *et al.* 2010), together raising the possibility that Mig3p has functionally diverged from Mig1p and Mig2p. Importantly, however, all of these studies have been performed in closely related laboratory strains.

Here we show that Mig3p plays a role in catabolite repression in a wild strain of yeast, YPS163, and an additional regulatory role upon ethanol exposure. Our results suggest that Mig3p function has been largely lost in the laboratory strain, possibly due to inadvertent laboratory selective pressures. These results have important implications for the glucose-signaling network in yeast.

## Materials and Methods

### Strains and growth conditions

Strains are listed in Supporting Information, Table S1. Deletions in the BY4741 background were obtained from Open Biosystems and verified by PCR. *MIG3* was deleted from a haploid derivative of YPS163 (YPS163-1 *hoΔ*::HygMX, referred to as the YPS163 parent or wild-type) (Lewis *et al.* 2010) by homologous recombination with the KanMX cassette amplified from the yeast knockout strain (Winzeler *et al.* 1999) and subsequently verified by diagnostic PCR. Diploid hybrid strains for reciprocal hemizygosity were generated by mating YPS163-1 *hoΔ*::HygMX *mig3Δ*::KanMX to BY4742 (AGY734), or YPS163-1 *hoΔ*::HygMX to BY4741 *mig3Δ*::KanMX (AGY680). Strains were grown either in YPD [1% (w/v) yeast extract, 2% (w/v) bactopeptone, 2% (w/v) glucose] medium or SC -Uracil medium containing 2% dextrose or 2% galactose as noted.

BY4741 cells harboring galactose-inducible, GST-tagged *BY*_*MIG3* (Open Biosystems) (Sopko *et al.* 2006) or the empty pEGH vector were grown in SC -Uracil with 2% galactose for ∼16 hr to induce *BY_MIG3* expression. To measure the resulting ethanol tolerance, cells were then exposed for 2 hr to multiple doses of ethanol as noted, at which time viability was measured by colony-forming units or LIVE/DEAD staining (Invitrogen, Carlsbad, CA) read on a Guava EasyCyte flow cytometer (Millipore, Billerica, MA) according to manufacturers’ instructions.

### Array hybridization and analysis

Expression in response to *MIG3* overexpression was assessed in biological triplicates by comparing RNA from cells harboring the GST-tagged *BY_MIG3* construct (pGST-*BY_MIG3*) with RNA from cells carrying the empty vector pEGH, grown in 2% galactose as described above. To measure the ethanol response, log-phase wild-type or *mig3Δ* cells of both the YPS163 and BY4741 backgrounds were exposed to 5% ethanol for 30 min. RNA collected from ethanol-treated cells was labeled and compared with RNA from the corresponding unstressed strain, in biological triplicates. Expression due to reciprocal hemizygosity was assessed by comparing RNA from each hybrid described above with a YPS163-1 *hoΔ*::HygMX reference, in biological duplicates.

Cell collection, RNA isolation, and cDNA labeling were performed as described (Berry and Gasch 2008; Gasch 2002), except that total RNA was labeled with a mixture of oligo-dT and random hexamer at a 1.7:1 molar ratio. Inverse dye labeling was used in replicates to control for dye-specific effects. Samples were hybridized to custom Nimblegen tiled arrays [previously validated for gene expression analysis in Lee *et al.* (2011) and Huebert *et al.* (2012)] spanning both strands of the yeast genome, according to the manufacturer’s instructions (Roche-Nimblegen). Arrays were scanned and analyzed with a GenePix4000 scanner (Molecular Devices, Sunnyvale, CA), and the signal from both channels was extracted with the program NimbleScan. Data normalization was performed using background subtraction followed by quantile normalization of the pooled arrays as in Wohlbach *et al.* (2011). Expression differences were taken as the log_2_ of the red/green signal from the arrays, except for the comparison of unstressed cells, for which the array signal corresponding to unstressed wild-type or *mig3Δ* cells was extracted from the ethanol arrays and normalized as described above. All microarray data are available through the NIH Gene Expression Omnibus (GEO) database under accession number GSE40153.

Genes with basal expression differences in mutant *vs.* paired wild-type samples were identified by comparing the array channels corresponding to unstressed cells, using the Bioconductor package limma v. 3.10.2 (Smyth 2004) and *q*-value FDR correction (Storey and Tibshirani 2003) (see File S1 for the limma output and the normalized gene expression values). Differences in the ethanol response were identified using limma by comparing the log_2_ fold-change in expression in wild-type and *mig3Δ* cells using a contrast matrix. For the duplicate reciprocal hemizygosity microarrays, genes with *P* < 0.05 (Smyth 2004) and whose mean expression was greater than 2 standard deviations from each other were called significant. Enrichment of gene ontology (GO) functional categories was performed using GO-TermFinder (http://go.princeton.edu/cgi-bin/GOTermFinder) (Boyle *et al.* 2004), with Bonferroni-corrected *P* < 0.01 taken as significant (see File S2). Motif analysis was performed using MEME (Bailey and Elkan 1994). Matches to the known Mig3-binding matrix (Badis *et al.* 2008) were scored in the 800 bp upstream region using RSA-tools matrix scan (http://rsat.ulb.ac.be/) (Turatsinze *et al.* 2008); enrichment was estimated with Fisher’s exact test, comparing with all genes in the genome with upstream matrix matches.

## RESULTS

### Mig3p affects yeast ethanol tolerance

**Figure 1:**
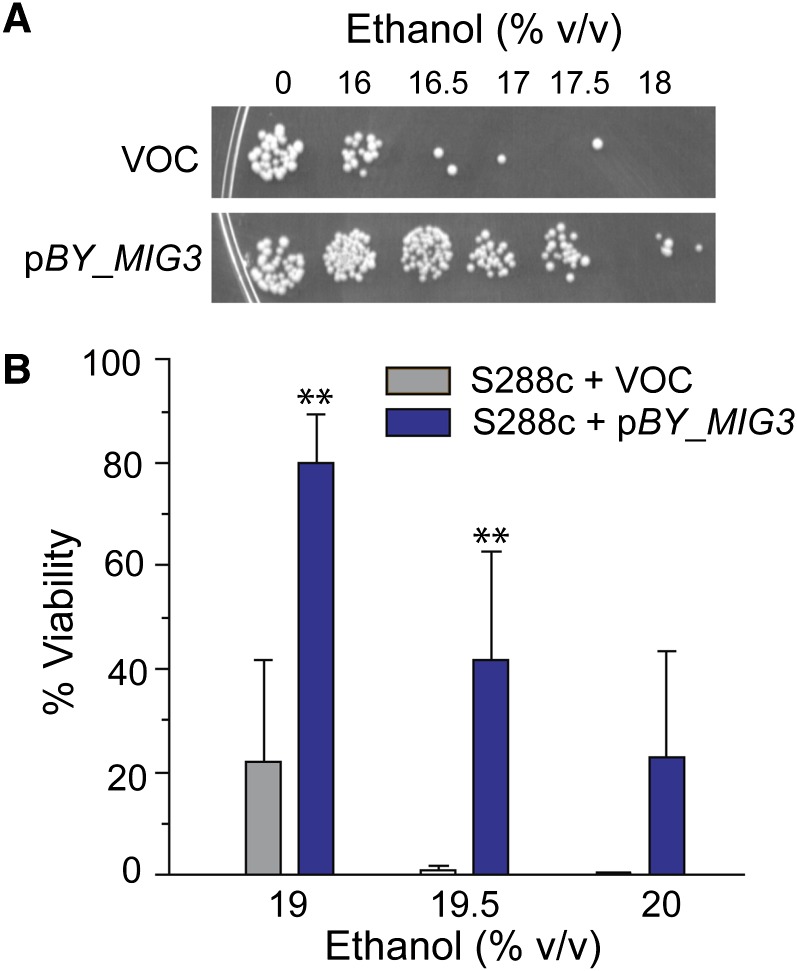
Mig3p overexpression increases ethanol resistance. *BY_MIG3* was cloned downstream of the *GAL1-10* promoter and overexpressed in S288c-derived strain BY4741. *BY_MIG3* was induced by growth on 2% galactose. Cells were exposed for 2 hr to increasing doses of ethanol before an aliquot of cells was plated to score viability. (A) Representative viability assays for cells harboring a vector-only control (VOC) or the *BY_MIG3* overexpression construct; ethanol doses to which cells were exposed are shown along the top. (B) As in (A), except viability was scored quantitatively using flow cytometry to determine the proportion of propidium iodide negative (*i.e.*, live) cells. Error bars represent standard deviation of biological triplicates. (**P < 0.01, *T*-test).

We previously studied the difference in ethanol-dependent genomic expression in wild yeast strains compared with an S288c-derived lab strain to identify new genes and processes involved in ethanol tolerance (Lewis *et al.* 2010). Of the thousands of genes with strain-specific differences in ethanol-dependent gene expression, *MIG3* stood out because it was a potential transcriptional regulator. *MIG3* was strongly repressed in the lab strain responding to ethanol but only slightly repressed in wild strains, such that final levels were in fact higher in the wild isolates than the lab strain (Lewis *et al.* 2010). To test its role in ethanol tolerance, we overexpressed the S288c allele of *MIG3* (*BY_MIG3*) in the lab strain and measured ethanol tolerance compared with an empty-vector control. Overexpression of *MIG3* produced a marked increase in ethanol resistance (Figure 1).

### Deletion of *MIG3* affects the expression of hundreds of genes in YPS163 but not the lab strain

To test for a possible regulatory role, we next measured global gene expression in the lab strain lacking *MIG3* relative to a wild-type control, using tiled, strand-specific genomic microarrays. Consistent with other studies (Lutfiyya *et al.* 1998; Westholm *et al.* 2008), there were no genes whose expression was affected at a false discovery rate (FDR) of 5% (File S1). This is in stark contrast to deletion of the related *MIG1* repressor from the lab strain, which in other studies was shown to affect the expression of ∼200 genes under standard growth conditions (Lutfiyya *et al.* 1998; Westholm *et al.* 2008).

**Figure 2:**
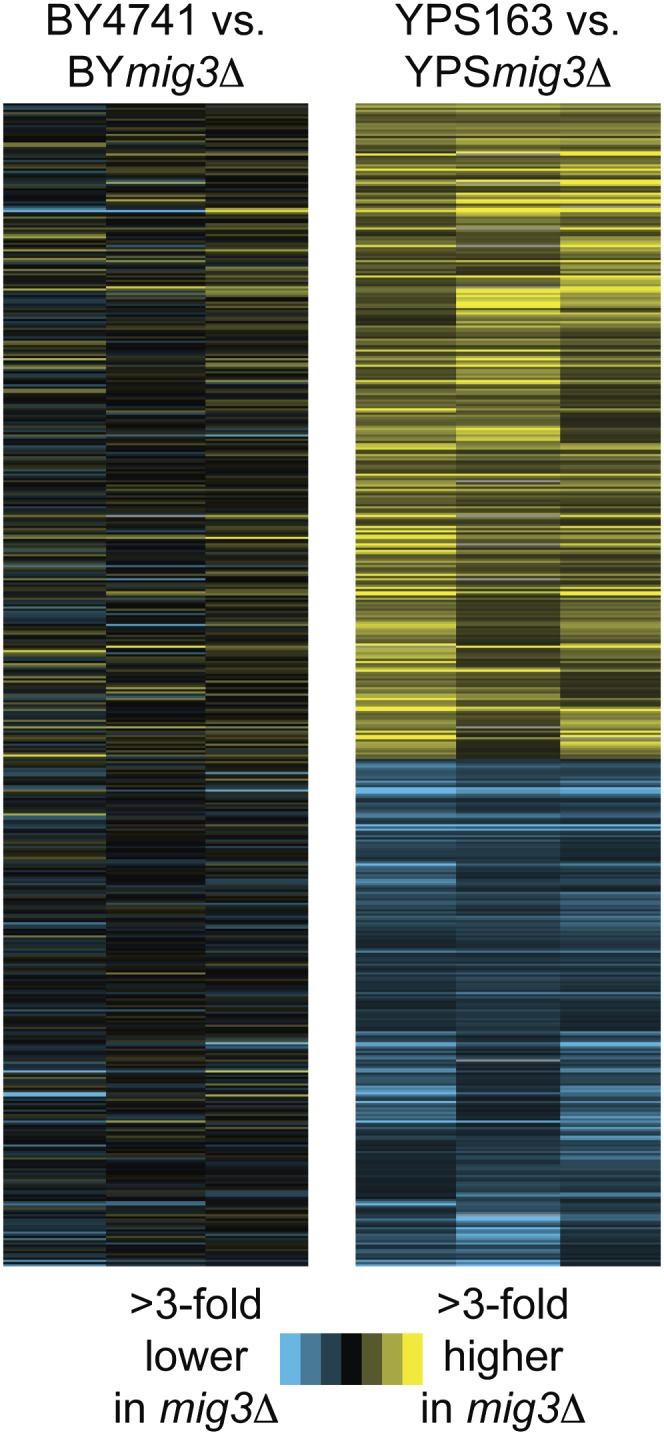
Mig3p affects basal gene expression in YPS163 but not BY4741. Hierarchical clustering of 865 genes affected by the lack of *MIG3* in YPS163 under standard conditions (FDR = 0.05). The heat map shows log_2_ expression differences between wild-type and *mig3Δ* cells in the BY4741 background (left) or YPS163 background (right) when cells are grown in unstressed glucose-containing medium. Genes are shown as rows, and each of triplicate experiments is shown as columns. Blue represents lower expression and yellow represents higher expression in the *mig3Δ* strain compared with its isogenic wild-type, according to the key.

We reasoned that the lack of mutant phenotype may be specific to the lab strain. We therefore measured genomic expression in the wild oak-soil strain YPS163 lacking *MIG3* relative to a wild-type control during unstressed growth on glucose-containing rich medium. In stark contrast to the lab strain, we found 489 genes that showed higher expression and 376 genes with lower expression in the YPS163 *mig3Δ* strain grown under standard conditions, compared with the isogenic parent (FDR = 0.05) (Figure 2 and File S1). Genes with higher expression in the *mig3Δ* strain were enriched for ribosomal proteins and unclassified genes (Bonferonni-corrected *P* < 0.01 in all cases). Additionally, many genes known to be repressed in the presence of glucose had higher expression in the *mig3Δ* strain, including genes involved in galactose metabolism (*GAL1*, *GAL2*, *GAL3*, *GAL4*, *GAL11*); maltose metabolism (*MAL32*, *IMA1*, *IMA2*); gluconeogenesis (*FBP1*, *FBA1*); hexose transport (*HXT9*, *HXT16*); and flocculation (*FLO1*, *FLO9*, *FLO10*). This gene set was enriched for known Mig1/2p targets identified in a previous study (Westholm *et al.* 2008) (*P* = 2 × 10^−4^, Fisher’s exact test) and was enriched for genes containing the known Mig binding site in the 800-bp upstream region (*P* = 3 × 10^−9^). Importantly, the expression of *MIG1* and *MIG2* was not affected in the *mig3Δ* strain, indicating that the effect cannot be simply explained through modulation of *MIG1/2* mRNA levels.

In contrast to these genes, genes with lower expression in the YPS163 *mig3Δ* strain showed little overlap with known Mig1p/Mig2p targets (Westholm *et al.* 2008) but were enriched for genes harboring Mig elements in the 800-bp upstream region (*P* = 4 × 10^−5^). This gene set was strongly enriched for genes involved in iron ion homeostasis and transport, in particular, amino acid and nitrogen transport (*P* = 3 × 10^−4^), organic acid transport (*P* = 4 × 10^−5^), and iron ion transport (7 × 10^−6^). Additionally, several genes involved in mitochondrial function or respiration had lower expression in the YPS163 *mig3Δ* strain (*Q0050*, *Q0115*, *CIT2*, *RSM18*, *YMR134W*, *SYM1*, *ALD5*, *COQ5*, *CAT5*, *MRPS18*, *MDM12*, *QCR10*, *COX2*, *COX3*).

**Figure 3:**
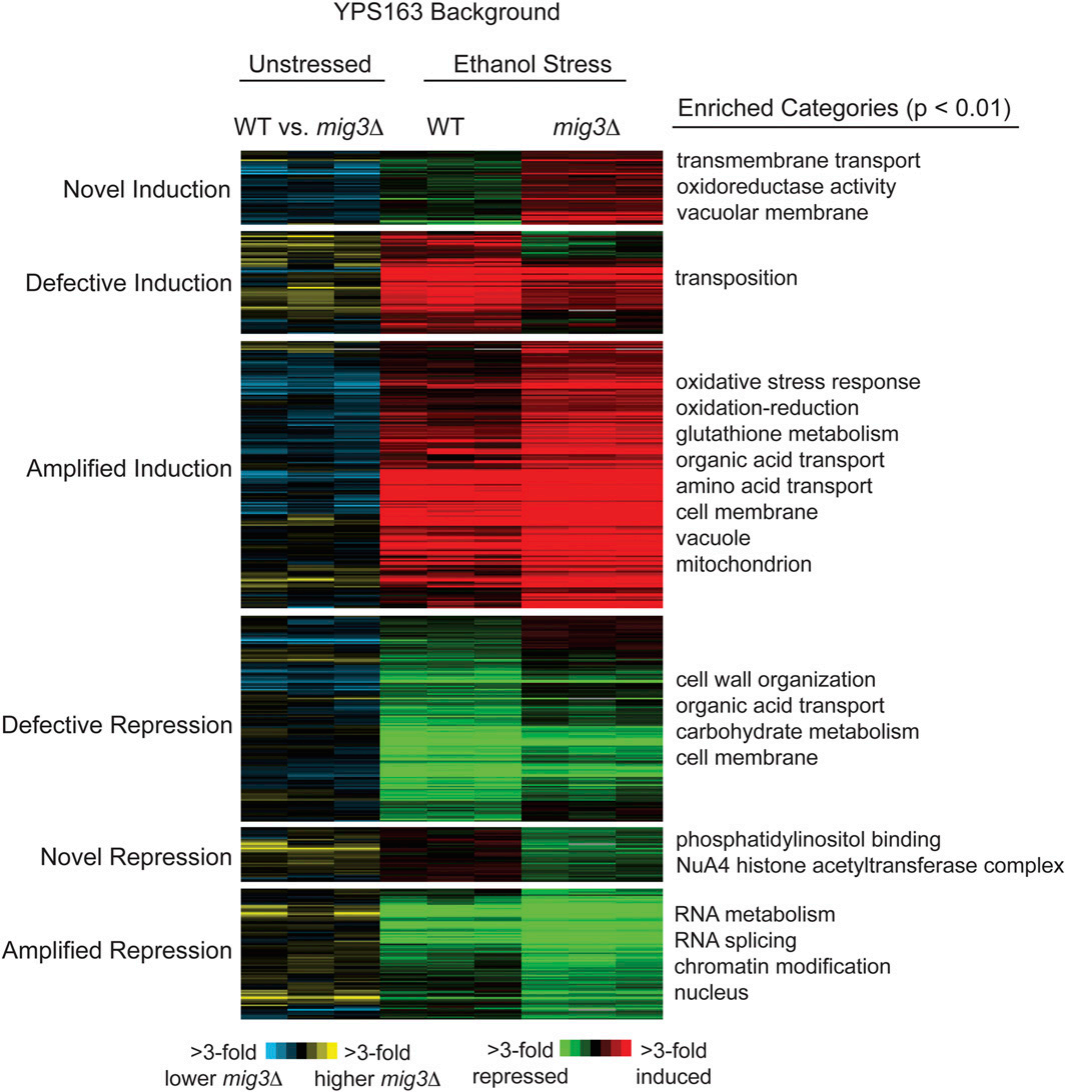
Genes dependent on YP_Mig3p for proper ethanol-responsive expression changes. Categorization and hierarchical clustering of 563 genes whose ethanol-responsive expression is affected by *MIG3* deletion in the YPS163 strain (FDR = 0.05). Left panels represent basal expression differences between wild-type YPS163 and the corresponding YPS163 *mig3Δ* strain (as in Figure 2), and right panels show the fold-change in expression 30 min after treatment with 5% ethanol, relative to unstressed cells, in wild-type YPS163 or the YPS163 *mig3Δ* mutant. Genes are shown as rows, and each of triplicate experiments is shown as columns. Red indicates gene induction and green represents gene repression upon ethanol treatment, according to the key.

Because Mig3p was implicated in ethanol tolerance, we also measured genomic expression in YPS163 *mig3Δ* responding to 5% ethanol for 30 min. We identified hundreds of genes with defective ethanol-responsive expression in the YPS163 *mig3Δ* strain compared with the YPS163 parent: 208 genes displayed weaker induction or repression relative to the wild-type parent, and 355 genes showed amplified expression changes upon ethanol treatment (FDR = 0.05) (Figure 3). Both groups showed significant overlap with genes displaying Mig3-dependent expression before stress, especially the group with amplified ethanol-responsive expression changes (*P* < 10^−4^). The latter group with hyper-responsive expression changes was enriched for oxidoreductases (*P* = 3 × 10^−8^), transporters (*P* = 5 × 10^−6^), and genes encoding proteins localized to membranes (*P* = 4 × 10^−12^) and to mitochondria (*P* = 1 × 10^−6^). Genes with smaller expression changes compared with the parental strain were also enriched for genes encoding membrane-localized proteins (*P* = 2 × 10^−14^), as well as genes involved in polysaccharide metabolism (*P* = 1 × 10^−9^), protein folding (*P* = 1 × 10^−7^), and metal ion homeostasis (*P* = 5 × 10^−6^).

We attempted to measure global Mig3p DNA binding through chromatin-immunoprecipitation (ChIP-seq), using a C-terminal myc-tagged Mig3p expressed in the YPS163 strain (Huebert *et al.* 2012; Longtine *et al.* 1998). However, the tagged construct did not fully complement *MIG3* deletion, and there were few bound loci not identified in unrelated ChIP experiments (J.A. Lewis and A.P. Gasch, unpublished data). Therefore, while the Mig3p-affected genes in YPS163 show clear enrichment for upstream Mig elements, we were unable to determine whether the genes are directly regulated by Mig3p (see *Discussion*).

### Partial loss of Mig3p function in the S288c-derived lab strain

Our results are consistent with the idea the S288c-derived lab strain displays a defect in Mig3p-dependent signaling, since there were no genes whose expression was affected by *MIG3* deletion in the lab strain, either under standard or ethanol-stress conditions (File S1). However, overexpression of the *BY_MIG3* allele clearly affected ethanol tolerance compared with the empty-vector control (Figure 1) and compared with other overexpression constructs tested that did not confer ethanol tolerance (Lewis *et al.* 2010). We further revealed that overexpression of *BY_MIG3* altered expression of 441 genes, 112 of which overlapped the Mig3p-affected genes defined in the YPS163 *mig3Δ* strain (*P* = 1 × 10^−10^). This suggests that Mig3p has retained some function in the lab strain despite the significant Mig3p-signaling defect.

The lab strain deficiency could be encoded at the *MIG3* locus or could result from *trans* effects elsewhere in the genome. To distinguish between these cases, we applied a reciprocal-hemizygocity approach. We created hybrid strains by mating BY4741 *mig3Δ* with YPS163 or by mating YPS163 *mig3Δ* with BY4742. The resulting hybrids are isogenic except for the single *MIG3* allele; therefore, any expression differences must be due to the *MIG3* locus. We found 85 genes with lower expression and 53 genes with higher expression in the hybrid strain harboring *BY_MIG3*. There was significant overlap between genes with differential expression in the BY_*MIG3* hybrid and Mig3p-dependent genes in the YPS163 *mig3Δ* strain (*P* = 3 × 10^−5^). Expression differences in the hybrid did not fully recapitulate the YPS163 *mig3Δ* effect (in part due to differences in statistical power) but confirm that at least part of the defect is due to sequence differences at the *MIG3* locus. As there was no difference in *MIG3* expression between the two strains, the gene expression differences between the hybrids are therefore likely due to functional differences between the Mig3p proteins.

This model was supported by expression QTL (eQTL) data of Smith and Kruglyak, who mapped expression differences between the vineyard strain RM11-1a and an S288c-derived lab strain (Smith and Kruglyak 2008). We partitioned progeny from the cross into two groups based on the marker closest to the *MIG3* locus, and then we identified the top 100 genes with significant expression differences between the two groups. We found significant overlap between genes with higher expression in the progeny with the *RM_MIG3* allele and Mig3p-dependent genes identified in YPS163 (*P* = 10^−4^). There was no difference in *MIG3* expression between the two progeny groups, consistent with the model that RM11-1a harbors a functional Mig3p, whereas the lab strain has partially lost Mig3p function.

**Figure 4:**
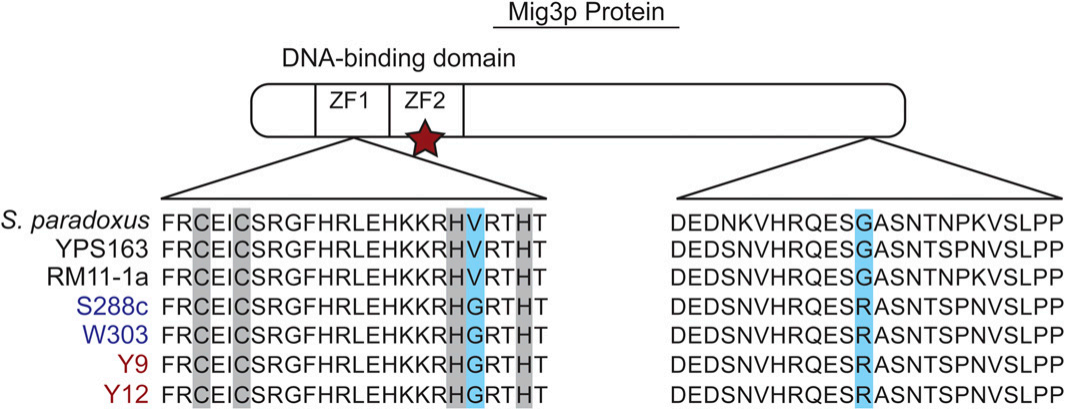
Local alignments of Mig3p implicate causal substitutions. Protein sequences surrounding S288c substitutions were locally aligned with Clustal W. ZF1 and ZF2 denote the first and second C_2_H_2_ zinc finger domains, respectively. The first alignment block highlights in blue the V36G substitution in ZF1, with the zinc-coordinating cysteine and histidine residues highlighted in gray. The second alignment block highlights the G364R substitution. Strain annotations are color-coded based on the Mig3p allele. The position of the premature stop codon (nonsense mutation) in Y9 and Y12 is highlighted with a red star. Diagram not to scale.

To implicate the causal substitutions, we compared Mig3p sequences from diverse *S. cerevisiae* strains (Doniger *et al.* 2008; Liti *et al.* 2009). Compared with both YPS163 and RM11-1a, BY_Mig3p contains only two substitutions, V36G and G364R. Residue V36 is right next to a zinc-coordinating histidine within the first C_2_H_2_ zinc finger in the DNA binding domain of Mig3p (Figure 4 and File S3) (Lutfiyya *et al.* 1998). Interestingly, several other strains of the 53 investigated harbor the V36G and G364R substitutions, including clinical isolate YJM451, Asian and African strains Y9 and Y12, respectively, and lab strain W303. Y9 and Y12 also contain a nonsense mutation leading to a premature stop codon at residue 50, which strongly suggests that the gene is nonfunctional in these strains. This information reveals that the likely causal polymorphisms—and the Mig3p defect—segregate in natural populations (see *Discussion*).

## DISCUSSION

The present study demonstrates the utility of using different strain backgrounds to probe cellular signaling and metabolism. By expanding our attention to wild strains, we uncovered a missing link in the yeast Mig network. All prior studies concluded that Mig3p plays at most a minor role in glucose signaling (Lutfiyya *et al.* 1998; Lutfiyya and Johnston 1996; Westholm *et al.* 2008). In contrast, we identified hundreds of Mig3p-affected genes in wild strain YPS163, providing new insights into the Mig network of catabolite repression. YP_Mig3p affected many classical catabolite-repressed processes, including alternative carbon source utilization and gluconeogenesis. This previously undescribed role for Mig3p is clearly not redundant with Mig1/2p function, since the single-gene deletion produced a major defect that could not be supplanted by the remaining Mig1/2p in the cell. This novel role for Mig3p in YPS163 warrants further investigation to distinguish the relative contributions of the Mig proteins to catabolite repression in wild yeast strains.

In addition to its novel role in catabolite repression, Mig3p has an additional function under ethanol stress. First, over 400 Mig3p-dependent genes were identified only under ethanol stress—these genes showed no significant expression defect in the YPS163 *mig3Δ* strain grown under standard conditions (Figure 3). A much smaller subset of genes (∼100) displayed Mig3-dependent expression under both standard and ethanol-stressed conditions. Second, overexpression of *MIG3* conferred ethanol resistance, supporting a functional role for Mig3p-affected genes in stress defense. Mig3p is known to be involved in arsenic resistance (Takahashi *et al.* 2010) and the DNA damage response (Dubacq *et al.* 2004) in laboratory strains. Thus, Mig3p may play distinct roles under various stressed and unstressed conditions in wild strains.

Mig3p could play a direct role in regulating many Mig3p-affected genes. Although we were unable to test direct binding through ChIP, the enrichment for upstream Mig elements in the Mig3p-affected genes is consistent with Mig3p regulation, especially for hyper-activated genes that are not regulated by Mig1/2p. If true, Mig3p could function as both an activator and repressor, since genes with an induction defect in the YPS163 *mig3Δ* strain were also enriched for genes with upstream Mig elements. This would be analogous to the proposed dual role of Mig1p (Lutfiyya and Johnston 1996; Santangelo 2006; Treitel and Carlson 1995).

It is also likely that many of the Mig3p-affected genes are indirect targets. Three quarters of Mig3p-affected genes did not harbor upstream Mig elements. Many of these could be targets of Mig3p-regulated genes. Indeed, over 40 genes encoding different transcription factors were differentially expressed in the YPS163 *mig3Δ* mutant, which likely explains a significant portion of the indirect effects. These transcription factors include those regulating metabolism (Nrg1p, Gal4p, Hap4p, Mot3p, Met4p, Met31p, Thi2p, Rgm1p), stress response (Msn4p, Cin5p), and meiosis (Xbp1p, Rme1p, Ume6p). This result hints at the potentially large amount of regulatory cross talk between Mig3p and other signaling pathways.

While we identified hundreds of Mig3p-affected genes in YPS163, our study, like several before ours (Lutfiyya *et al.* 1998; Westholm *et al.* 2008), failed to identify any Mig3p-affected genes in S288c. We hypothesize that S288c has largely lost Mig3p function through changes to the coding sequence. Lab strains are notably highly proliferative on rich media but grow relatively poorly on respiratory carbon sources (Gerke *et al.* 2006; Warringer *et al.* 2011). The S288c lab strain also has a major defect in ethanol response compared with other strains (Lewis *et al.* 2010). It is possible that laboratory passage on rich media has either rendered Mig3p dispensable or led to selective loss of function. In a previous study, we identified hundreds of genes that were differentially expressed between the S288c and YPS163 under both standard and ethanol-stress conditions (Lewis *et al.* 2010). There is significant overlap in genes with higher expression in both the YPS163 *mig3Δ* strain and S288c *vs.* YPS163 (*P* = 0.004), as well as for genes with lower expression in both the YPS163 *mig3Δ* strain and S288c *vs.* YPS163 (*P* = 9 × 10^−4^). This suggests that a subset of the gene expression differences between S288c and YPS163 under standard conditions can be explained through natural variation in Mig3p signaling.

Through sequence analysis, we identified a SNP in *BY_MIG3* that leads to a nonconservative V36G substitution. This substitution sits rights next to the first C_2_H_2_ zinc finger in the DNA-binding domain of Mig3p, which could conceivably affect Mig3p function. This SNP was identified in only 4 out of 53 *S. cerevisiae* strains. Intriguingly, two of those are wild strains that harbor the V36G substitution, as well as a premature stop codon at residue 50 that likely produces full loss of Mig3p function. Pseudogenization could have occurred subsequent to the V36G substitution due to loss of evolutionary constraint. Regardless, these data reveal that the polymorphism is segregating at low levels in *S. cerevisiae* populations. It will be interesting to assess the variation in Mig3p function in natural strains. This perspective will likely continue to shed light on important physiological processes that differ across genetic backgrounds.

## Supplementary Material

Supporting Information
