# Supplementary material for: Natural Variation in the Yeast Glucose-Signaling Network Reveals a New Role for the Mig3p Transcription Factor
Source: G3 (Bethesda). 2012 Dec 1;2(12):1607–12. doi: 10.1534/g3.112.004127 (PMC3516482; doi:10.1534/g3.112.004127)
Supplement: Supporting Information [file supp_2_12_1607__index.html]

Supporting Information 

# Natural Variation in the Yeast Glucose-Signaling Network Reveals a New Role for the Mig3p Transcription Factor

## Supporting Information for Lewis and Gasch, 2012

**Files in this Data Supplement:**

- Table S1 - Strains used in this study (PDF, 56 KB)
- File S1 - Microarray data and statistics (.xlsx, 3 MB)
- File S2 - GO enrichments (.xlsx, 95 KB)
- File S3 - Mig3p ClustalW Alignments (.txt, 29 KB)
