# Supplementary material for: Natural Variation in the Yeast Glucose-Signaling Network Reveals a New Role for the Mig3p Transcription Factor
Source: G3 (Bethesda). 2012 Dec 1;2(12):1607–12. doi: 10.1534/g3.112.004127 (PMC3516482; doi:10.1534/g3.112.004127)
Supplement: Supporting Information [file supp_2.12.1607_TableS1.pdf]

Table S1 Strains used in this study

Strain	Background	Group	Description	Source	Ploidy
DBY8268	S288c derivative	Lab	S288c derivative (*ura3-52/ura3Δ-0 ho/ho GAL2/GAL2*)	David Botstein	diploid
BY4741	S288c derivative	Lab	S288c derivative (*MATa ura3Δ-0 leu2Δ-0 met15Δ-0 his3Δ-1 ho*)	Jasper Rine	haploid
YKO0160	S288c derivative	Lab	S288c derivative (*MATa ura3Δ-0 leu2Δ-0 met15Δ-0 his3Δ-1 ho mig3Δ::KanMX*)	Open Biosystems	haploid
YPS163	wild strain	Oak	YPS163	Paul Sniegowski	diploid
AGY338	YPS163	Oak	YPS163 derivative (*MATa hoΔ::HygMX*)	this study	haploid
AGY733	YPS163	Oak	YPS163 derivative (*MATa hoΔ::HygMX mig3Δ::KanMX*)	this study	haploid
AGY680	BY_mig3Δ/YPS_MIG3 hemizygote	hybrid	YPS163/S288c hemizygous strain (*BY_mig3Δ/YPS_MIG3*)	this study	diploid
AGY734	YPS_mig3Δ/BY_MIG3 hemizygote	hybrid	YPS163/S288c hemizygous strain (*YPS_mig3Δ/BY_MIG3*)	this study	diploid
